# The complete chloroplast genome of *Phyllostachys edulis* f. *curviculmis* (Bambusoideae): a newly ornamental bamboo endemic to China

**DOI:** 10.1080/23802359.2021.1888663

**Published:** 2021-03-16

**Authors:** Li-Qin Gao, Yong-Long Li, Wen-Gen Zhang, Guang-Yao Yang

**Affiliations:** a Jiangxi Provincial Key Laboratory for Bamboo Germplasm Resources and Utilization, Forestry College, Jiangxi Agricultural University, Nanchang, P. R. China; b Nanchang Business College, Jiangxi Agricultural University, Gongqingcheng, P. R. China

**Keywords:** *Phyllostachys*, *Phyllostachys edulis*, *Phyllostachys edulis* f*. curviculmis*, chloroplast genome, phylogenetic relationship

## Abstract

*Phyllostachys edulis* (Bambusoideae) is a temperate woody bamboo with a long history of cultivation in China. *Phyllostachys edulis* f. *curviculmis* is the latest new forma that repored in 2018. Here, we performed the complete chloroplast genomes of *P. edulis* and *P. edulis* f. *curviculmis* using genome skimming. The length of two chloroplast genomes was 139,678 bp, and their GC contents were 38.9%. The sequences of each species contained 132 unique genes, including 39 tRNA, eight rRNA, and 85 protein-coding genes. Moreover, in subspecies-level, *P. edulis* ‘Pachyloen’ and *P. edulis* f. *curviculmis* are identical to *P. edulis* in the terms of chloroplast genome size, structure, and composition, further indicating their affinity.

*Phyllostachys edulis* (Carriere) J. Houzeau f. *curviculmis* H. X. Wang & J. S. Peng, a new form of moso bamboo recently found in Feng-Xin County of Jiangxi, China, has potentially ornamental value for the garden because of its uniquely apparent morphology with the S-shaped culm in the subfamily Bambusoideae (Poaceae) (Wang et al. 2018). The new forma, obviously different from other varieties of *P. edulis* (Ma et al. 2014), may play an important role in understanding the habit of erect growth and the diversity evolution of bamboos. However, the genetic information of the genus *Phyllostachys* is still scarce. Here, by the second-generation genome sequencing, the complete chloroplast genome of *P. edulis* f. *curviculmis* was firstly obtained, to expedite the study on the molecular phylogeny of *Phyllostachys*.

The total chloroplast genome DNA of *P*. *edulis* f. *curviculmis* was extracted from silica-dried fresh leaves, which were collected from the type location (Liu-Xi Village of Jiangxi, 28°40′18″N, 114°52′38"E). *P*. *edulis* collected in the bamboo garden of Jiangxi Agricultural University (Nanchang of China, 28°45′40″N, 115°49′31″E) using the same sampling method as *P*. *edulis* f. *curviculmis*. The voucher specimens of *P*. *edulis* f. *curviculmis* and *P*. *edulis* (accession number Gao1105 and Gao1108, JXAU!) were deposited at the herbarium of the College of Forestry, Jiangxi Agricultural University, China. Illumina paired-end (PE) library was prepared and sequenced in Kunming Institute of Botany, Chinese Academy of Sciences (CAS), and all contigs of the chloroplast genome sequence were spliced and assembled by using SPAdes 3.13.0 and Geneious 9.0.5 (http://www.geneious.com/). Then, the complete chloroplast genome was annotated using CPGAVAS2 (Shi et al. 2019), and simple sequence repeats (SSR) were detected by MISA (http://pgrc.ipk-gatersleben.de/misa).

The complete chloroplast genome sequences of *P*. *edulis* f. *curviculmis* (MW007169) and *P*. *edulis* (MW007170) were similar with their total length and gene contents. Their chloroplast genome was 139,678 bp in length, including a typical quadripartite structure with two inverted repeats (IRs) of 21,798 bp separated by a large single copy (LSC) of 83,212 bp and a small single copy (SSC) of 12,870 bp. A total of 132 genes in the chloroplast genome, including 85 protein-coding genes, 8 ribosomal RNA (rRNA) genes, and 39 transfer RNA (tRNA) genes, are identified, and 34 SSR sites are detected in its cp genome. Protein-coding regions (CDs) contribute 41.53% of the chloroplast genome, and the total of GC content is 38.9%.

To reconstruct the phylogenetic tree of the trib. Arundinarieae, 32 previously published plastomes of the tribe and 3 species as outgroup were downloadedfrom the NCBI. The maximum-likelihood phylogenetic tree was performed by using RAxML 8.2.8 (Stamatakis 2014), and Bayes tree was performed by using MrBayes 3.2.6 (Ronquist and Huelsenbeck 2003). Phylogenetic analysis showed that it was consistent with the previous results (Huang et al. 2020; Zhang et al. 2019; Zheng et al. 2020). All samples of *Phyllostachys* were grouped together into one well-supported clade (i.e. *Phyllostachys* clade) (Figure 1), and *P*. *edulis* f. *curviculmis* was gathered with other moso bamboo taxa.

**Figure 1.**
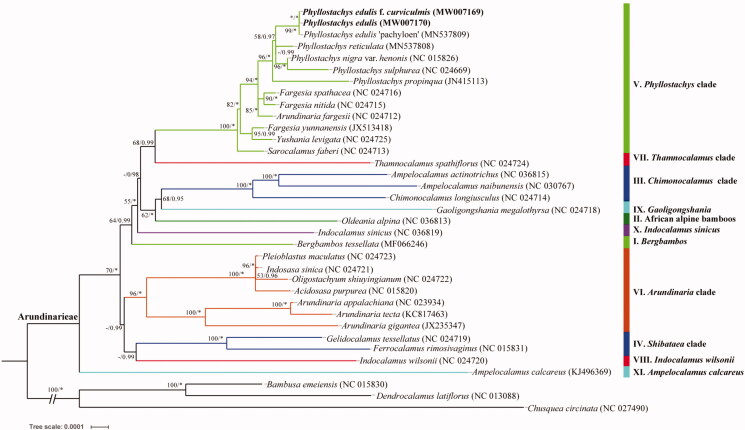
Maximum-likelihood tree inferred from 35 woody bamboo chloroplast genomes. Colored branches indicate the 11 Arundinarieae lineages (I–XI). Numbers associated with branches are ML bootstrap values, and Bayesian posterior probabilities, respectively. Asterisks indicate 100% bootstrap support or 1.0 posterior probability. Hyphens indicate the bootstrap support or posterior probability lower than 50% or 0.5.

## Data Availability

The genome sequences data that support the findings of this study are openly available in GenBank of NCBI at (https://www.ncbi.nlm.nih.gov/) under the accession no. MW007169-MW007170. The associated BioProject, SRA, and Bio-Sample numbers are PRJNA689975, SUB8843788, and SAMN17224149, SAMN17224150, respectively.
